# The Long-Term Effects of Mandibular Distraction Osteogenesis on Developing Deciduous Molar Teeth

**DOI:** 10.1155/2012/913807

**Published:** 2012-10-17

**Authors:** Paul Hong, Elise Graham, James Belyea, S. Mark Taylor, Donald B. Kearns, Michael Bezuhly

**Affiliations:** ^1^Dalhousie Pediatric Craniofacial Group, Department of Surgery, IWK Health Centre, Dalhousie University, P.O. Box 9700, Halifax, Nova Scotia, Canada B3K 6R8; ^2^Division of Otolaryngology-Head and Neck Surgery, Department of Surgery, Dalhousie University, Halifax, Nova Scotia, Canada B3K 6R8; ^3^Division of Pediatric Otolaryngology, Rady Children's Hospital, University of California-San Diego, San Diego, CA 92123, USA

## Abstract

*Background.* Many studies have demonstrated the effectiveness of mandibular distraction osteogenesis (MDO) in alleviating the micrognathia-associated upper airway obstruction but very few studies have focused on long-term dental outcomes. *Objective.* To report the effect of MDO on developing deciduous molars in the distraction area. *Methods.* A retrospective chart review was performed to identify patients with Pierre Robin sequence who underwent MDO with documented long-term dental assessments. *Results.* Ten children (mean age at surgery 69.8 days; 6 boys and 4 girls) were included for analysis. All patients underwent bilateral MDO with an inverted L-shaped osteotomy to avoid injuring tooth buds. The dental developmental stage was primary dentition in all children. Overall, 3 patients developed minor dental problems involving 4 molar teeth (2 root malformations and 2 shape anomalies) but they did not require any interventions. *Conclusion.* Significant primary molar developmental complications were not seen in our patients. The use of internal distractor device with an inverted L-shaped osteotomy seems to be a safe surgical approach in regards to dental outcomes.

## 1. Introduction

Upper airway obstruction secondary to micrognathia was first widely described by P. Robin in 1934 [1]. He described a constellation of findings, which included micrognathia, glossoptosis, and in some patients, cleft palate. These findings are now commonly referred to as Pierre Robin sequence (PRS). Some craniofacial syndromes were later recognized to be associated with PRS. Most notably they include Stickler syndrome, Treacher Collins syndrome, and Nager syndrome [2].

Micrognathia can cause upper airway obstruction due to posterior tongue collapse and physical obstruction of the oropharyngeal and hypopharyngeal regions. Although the majority of children born with micrognathia or PRS can be treated with conservative management, some patients may have significant respiratory issues, necessitating more aggressive interventions [1, 3, 4].

Traditionally, tracheostomy has been the most effective and definitive treatment option for these patients [5]. Tracheostomy, however, is associated with frequent morbidity, high cost, and occasional mortality [6–8]. 

Mandibular distraction osteogenesis (MDO) is a relatively new treatment option in children with PRS, which has been shown to be very effective in relieving the upper airway obstruction by gradually lengthening the mandible. Overall, MDO is considered to be safe with low incidence of major complications [9, 10]. Some of the potential risks include marginal mandibular nerve paralysis or paresis, inferior alveolar nerve injury, soft-tissue or bone infections, device failure or migration, scarring, poor healing of regenerate bone, and dental complications [11, 12]. Although considered a relatively minor disadvantage, long-term dental injuries are underrecognized and have not been well studied [12, 13]. 

In the present paper, the effects of MDO on deciduous dental development are reported in a series of infants with PRS. More specifically, the long-term health status of mandibular molar teeth with dental and radiographic assessments is presented.

## 2. Methods

### 2.1. Patients

An institutional review board approval was obtained for this paper.

A retrospective chart review was performed to identify all children who underwent bilateral MDO with complete long-term (at least 3-years followup) dental assessments. Consultation with the pediatric craniofacial team was carried out in all children. Patient characteristics, operative details, complications, and postoperative dental outcomes were documented. More specifically, the long-term changes noted with the mandibular molar dentitions with dental radiographs and pediatric dental assessments were reviewed for each patient. The pediatric dentists' report was the primary outcome measure utilized and if there was any uncertainty regarding the reports, it was reviewed again with the aid of a pediatric dentist. 

### 2.2. Distraction Osteogenesis Surgery

**Figure 1:**
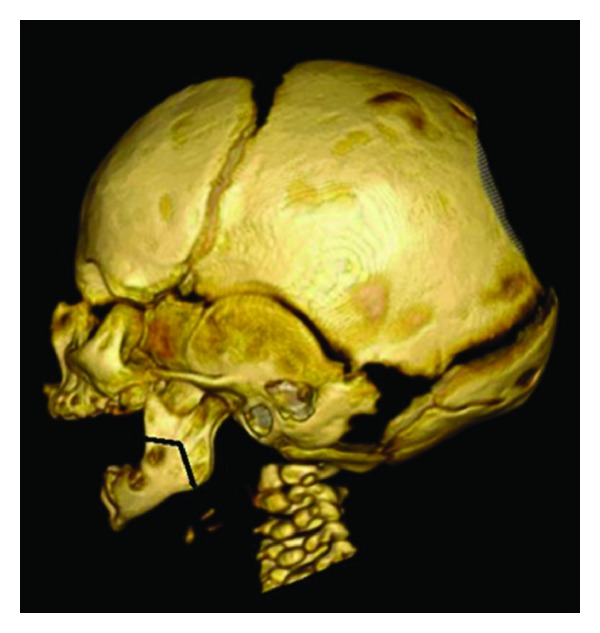
A lateral view of the three-dimensional reconstructed computed tomographic image of an infant with micrognathia-associated upper airway obstruction. Note the inverted L-shaped osteotomy design on the left mandible to avoid the tooth buds. The internal distractor device footplates are secured on the lower aspect of the osteotomy.

All patients were placed under general anesthesia and intubated orally. Internal mandibular distractor devices were used in all patients. One patient had an absorbable distractor device placed through an intraoral incision while the rest had nonabsorbable devices with an external cutaneous approach (modified Risdon incision). An inverted L-shaped buccal corticotomy (including the cephalic and caudal borders) was first performed with a reciprocating saw. The remainder of the osteotomy was performed with an osteotome placed along the lingual cortex. The inverted L-shaped osteotomy was designed based on the preoperative imaging to avoid the tooth buds (Figure 1). The imaging modality consisted of computed tomograms with three-dimensional reconstructions. As well, with the completion osteotomy technique using the osteotome along with the lingual aspect, the inferior alveolar nerve was identified and preserved in all cases. The vector of distraction in each case was planned during the preoperative phase using clinical and imaging assessments.

Distraction was initiated on the first postoperative day at a rate of 1.0 mm to 2.0 mm per day. In general, the neonates were distracted 1.0 mm twice per day, while infants were distracted 0.5 mm twice per day. Active distraction was ended when there was no longer maxillomandibular discrepancy but the distractor device (nonabsorbable) was left in place for the consolidation period, which lasted between 6 and 8 weeks.

## 3. Results

Ten children were identified who underwent bilateral MDO and who had complete documentation of long-term dental assessments (range 3–5 years). Six boys and four girls underwent surgery at a mean age of 69.8 days with a range of 25 to 102 days. All patients were found to have PRS (micrognathia, glossoptosis, and upper airway obstruction) and eight also had cleft palate. Half of the patients had associated genetic syndromes (Table 1). The dental developmental stage was primary dentition in all patients.

All patients had clinical symptoms and physical examination findings of severe upper airway obstruction that was not adequately managed with conservative therapy. Half of the patients required intubation prior to the distraction procedure to secure the airway. Briefly, newborns were considered to have severe airway obstruction requiring surgical intervention if they were intubated at birth and later failed extubation and/or presented with significant oxygen desaturations with signs of respiratory distress despite conservative measures, such as positioning. More specifically, those children who failed extubation attempts, temporary airway placements with nasopharyngeal or oropharyngeal trumpets, and continuous positive airway pressure therapies were considered to be surgical candidates. Moreover, pulse-oximetry levels less than 90% for 30 seconds or greater as well as pCO_2_ greater than 50 mm Hg on blood gases were also considered as preoperative indicators. 

None of the patients had any significant complications related to the mandibular distraction surgery. Two patients had local erythema and tenderness around the activator site, which was treated successfully with antimicrobial ointment and systemic antibiotic therapy. There were no cases of facial nerve injury, device failure, or significant scarring. The range of distraction distances was from 14 to 20 mm (mean 16.8 mm).

**Figure 2:**
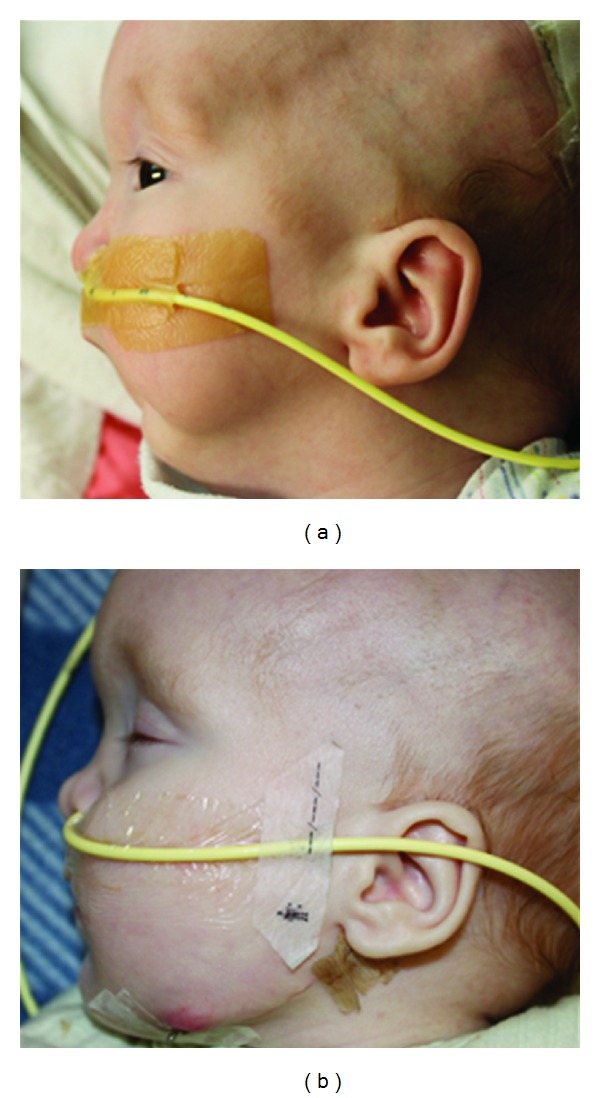
Photographs of an infant with micrognathia and upper airway obstruction before (left) and after (right) mandibular distraction osteogenesis. Note the change in the profile of the lower face (permission to use the photograph was granted from the caregiver).

All patients were able to avoid a tracheostomy and other airway interventions, including supplemental oxygen, after the completion of the distraction phase of the procedure (Figure 2). No patients required any monitoring or other home care measures on discharge from the hospital with regards to upper airway obstruction.

All patients were seen on a regular basis in a multidisciplinary craniofacial surgery clinic. Specifically, the craniofacial skeletal assessments were performed by the reconstructive surgeon, oral and maxillofacial surgeon, and the orthodontist; the dental assessments were conducted by the pediatric dentist and the orthodontist. These assessments mainly involved clinical examinations and dental panoramic radiographs were obtained as per the order of the pediatric dentist. Long-term followup assessments revealed no clinically significant relapses that required repeat MDO procedure or other airway interventions. Four patients did have some degree of retrognathia with class II malocclusion but they were not considered significant and did not cause any problems. Seven children had non-MDO-related dental problems, including poor hygiene, dental carries and decay, and anterior lower crowding. 

Overall, the mandibular molars considered to be affected by MDO were found in 3 of 10 patients with 4 teeth being involved (Table 1). The changes mainly included structural root malformations (*n* = 2) and positional changes (*n* = 2). More specifically, the root malformation involved primary second molar teeth in both cases, and the positional changes were reported as minor rotations. Finally, one of the molars with root malformation also had a minor shape deformity (micropits). They were all considered nonsignificant, and none required any interventions. Furthermore, all primary molar buds located in the distraction region erupted without any complications.

Other dental problems that may be related to MDO, such as the destruction of tooth follicles or hindered dental development, were not observed. As well, no other odontogenic complications, such as dentigerous cyst formation, were noted. 

## 4. Discussion

Traditionally, MDO was used in older children but more recently, many authors have performed the procedure in younger children, including neonates and infants to relieve severe upper airway obstruction and at the same time obviate the need for a tracheostomy [10, 14]. Many of the newborns that undergo MDO will not have any recognizable dental structures on intraoral examination. However, there is a real opportunity for the tooth buds to be injured by either the osteotomy and/or screw or pin placement. Furthermore, there is also a risk of tooth migration that may occur with the application of the distraction forces. It is important, therefore, to examine the long-term consequences of primary dental development in young children that undergo MDO. 

To date, only a few studies have addressed dental complications associated with MDO. Moreover, long-term dental outcomes seem to be an underrecognized and underreported entity. For example, in a large review of MDO complications, tooth damage was noticed in only 1 of 589 cases [11]. In another study where questionnaires were sent to reconstructive surgeons, mandibular dental injury was reported to occur in only 2% of patients [13].

Interestingly, when the sole focus of a study was dental outcomes, the associated risk has been reported to be much higher. For instance, mandibular molars were affected by MDO in 13 of 17 patients in a report by Kleine-Hakala et al. [15]. Furthermore, significant complications, such as the destruction of tooth follicles and failed eruption of molars, were observed. Another series reported normal molar development in less than half of the time after MDO procedure [16]. Specifically, there was distalization and perforation of dental buds, shape deformities, and dental root injuries that lead to absorption. As well, there are reports of other odontogenic complications related to MDO, such as the development of dentigerous cysts [16, 17]. One case of dentigerous cyst occurred after an osteotomy was placed across the tooth follicle, which resulted in fibrous union and failure of osteogenesis [17]. 

 Unlike the abovementioned studies [15, 16], our series had a relatively low rate of dental complications. In addition, the molar anomalies were considered minor and did not require any intervention, other than monitoring. The differences most likely stem from the dissimilar dates of the studies and the varying surgical techniques utilized. Specifically, the studies with more serious and greater complication rates were published several years ago. As well, the older surgical technique and the distractor devices used were different.

 The major causes of dental damage appeared to be associated with poorly planned osteotomies and/or placement of screws or pins where vital dental structures exist. For instance, several cases of dental damage were reported to result from splitting of the tooth follicle during the mandibular osteotomy [15]. This can be avoided by using an inverted L-shaped osteotomy as illustrated in Figure 1. This technique places the cut line higher in the ramus and is inclined obliquely from the buccal to the lingual region [18].

Traditionally, external mandibular distractor devices were used more commonly by reconstructive surgeons [10]. However, these devices have been associated with pin-site facial scarring and were considered to be cumbersome and intimidating by intensivists and caregivers [10]. Furthermore, many previous dental injuries seemed to be related to the use of bicortical fixation of external distractor pins [15]. Although external distractors have the advantage of multivector advancements and do not require a second operation for its removal, internal distractor devices have been becoming more popular [10]. In the present series, only internal distractor devices were utilized, which may have reduced the incidence of molar injuries. They do not involve bulky transfacial pins that may injure dental buds. Since the current technology allows suitable sized internal distractor devices to be used in newborns, the dental complications should correspondingly be reduced over time.

Some authors have reported successful outcomes with absorbable internal distraction devices, which were also used in one of the patients in the current series [10]. However, the long-term experience is limited with this type of device at this time. 

 Some of the limitations in the current report include the small sample size and the retrospective nature of the data collection. As well, more than one pediatric dentist performed the consultations, and only deciduous teeth development was analyzed. Finally, some of the observed dental findings may be related to the PRS or the associated syndrome itself.

## 5. Conclusion

 Although high rates of dental complications have been reported in the past, the use of the newer distractor devices with improved planning of mandibular osteotomies may result in reduced dental complications.

## Figures and Tables

**Table 1:** Summary of the patient characteristics and dental outcomes.

Patient	Age* (Days)	Gender	PRS^†^	Syndromes	Dental complications
1	89	M	Yes	Otopalatodigital	Minor positional changes
2	25	F	Yes	Stickler	None
3	94	M	Yes	4p deletion	Minor root malformation
4	78	M	Yes	No	None
5	32	M	Yes	No	None
6	69	F	Yes	Stickler	Minor root malformation and shape deformity Minor positional changes
7	56	M	No CP	No	None
8	87	M	Yes	No	None
9	66	F	Yes	No	None
10	102	F	No CP	Hemifacial microsomia	None

^∗^Age at the time of operation.

^†^Pierre Robin sequence (micrognathia, glossoptosis, and cleft palate).

CP: cleft palate.
